# Supplementary material for: Low level of antioxidant capacity biomarkers but not target overexpression predicts vulnerability to ROS-inducing drugs
Source: Redox Biol. 2023 Feb 23;62:102639. doi: 10.1016/j.redox.2023.102639 (PMC10053401; doi:10.1016/j.redox.2023.102639)
Supplement: Multimedia component 1 [file mmc1.pdf]

Supplementary Table S1: Chemical compounds

**Chemical compound**	**Company**	**Catalog No**
1S,3R-RSL 3	Sigma-Aldrich	SML2234
Auranofin	Sigma-Aldrich	A6733-10MG
Azacytidine (AZA)	Sigma-Aldrich	A2385
BAY11-7082	Sigma-Aldrich	196870
BIX-01294	Sigma-Aldrich	B9311
Blasticidin	Santa Cruz	sc-495389A
BML-210	BIOTREND CHEMIKALIEN	B5968
Bortezomib	New England Biolabs	2204 S
BRD-6929	Sigma-Aldrich	SML2521
BSA	Serva	11926
C188-9	Merck	44915076
Carboxy-PTIO	Sigma-Aldrich	C221
CBB-1007	Sigma-Aldrich	489478
CPUY192018 (CPUY)	Biotrend	AOB9974-1
Cyclodextrin (CD)	Biosynth	OC15979
Dimethylsulfoxide (DMSO)	Sigma-Aldrich	D2650
DKFZ-682	produced by AG Gunkel/Miller, DKFZ	
Erastin	Cayman Chemical	17754
Ferrostatin-1	Sigma-Aldrich	SML0583
GSK-J4	Sigma-Aldrich	SML0701
H_2O_2	Sigma-Aldrich	H1009
LCS-1	Sigma-Aldrich	SML0466
Methylstat	Sigma-Aldrich	SML0343
MG-132	Sigma-Aldrich	474790
ML162	Cayman Chemical	20455
ML210	Sigma-Aldrich	SML0521
Necrosulfonamide	Sigma-Aldrich	480073
NOC-18	Santa Cruz Biotechnology, Inc.	sc-202247
PAOA	BIOMOL	Cay22942
Piperlongumine	Merck	528124
PRIMA-1	MedChem Express	HY-19980A
Puromycin	Sigma-Aldrich	P9620
PX-12	Sigma-Aldrich	M5324
SC75741	Sigma-Aldrich	SML2382
STAT3-IN-1	Hölzel	HY-100753
TPCA-1	Sigma-Aldrich	T1452
UNC-0642	Sigma-Aldrich	SML1037
WP1130	BIOTREND CHEMIKALIEN	A8323
YM-155	Sigma-Aldrich	574662

Supplementary Table S2: Primary and secondary antibodies

Antigen	Product / Company	Species	Dilution WB
PRDX1	8499/ Cell Signaling	rabbit	1:1000
PRDX3	ab128953/ Abcam	rabbit	1:2000
GCLM	HPA023696/ Sigma-Aldrich	rabbit	1:100
NRF2	ab62352/ Abcam	rabbit	1:1000
xCT/SLC7A11	12691/ Cell Signaling	rabbit	1:1000
GSR	ab124995/ Abcam	rabbit	1:1000
AKR1C3	A6229/ Sigma-Aldrich	mouse	1:500
UGDH	SAB 4503060/ Sigma-Aldrich	rabbit	1:750
PTGR1	HPA036724/	rabbit	1:250
TXN	ab133524/ Abcam	rabbit	1:10000
CBR1	HPA018433/ Atlas Antibodies	rabbit	1:750
PGD	HPA031314/ Sigma-Aldrich	rabbit	1:500
BLVRB	HPA041698/ Sigma-Aldrich	rabbit	1:100
STAT1	14994/ Cell Signaling	rabbit	1:1000
pSTAT (Y701)	7649/ Cell Signaling	rabbit	1:1000
STAT3	9139/ Cell Signaling	mouse	1:1000
pSTAT3 (Y705)	9145/ Cell Signaling	rabbit	1:1000
STAT5	25656/ Cell Signaling	rabbit	1:1000
pSTAT5 (Y694)	9359/ Cell Signaling	rabbit	1:1000
Cas9	14697T/ Cell Signaling	mouse	1:1000
GAPDH	sc-365062/ Santa Cruz	mouse	1:1000
Lamin A/C	2032/ Cell Signaling	rabbit	1:1000
monoclonal anti β-tubulin	T0198/ Sigma-Aldrich	mouse	1:1000
IRDye 680LT antimouse IgG	926-68022/ LI-COR	donkey	1:5000
RDye 680LT antirabbit IgG	926-68023/ LI-COR	donkey	1:5000

		Correlation with EC50 DKFZ-682				
	distance CTRP	pilot (6 cell lines#)	expanded panel	Number of cell lines tested	Selectivity*	ROS induction**
DKFZ-682	0	1	1	31	13	>2
Necrosulfonamide	1.09	0.98		6 #		>17
Methylstat	1.28	0.79	0.6	24 §	8	>3
1S3R-RSL3	1.37	0.89		6 #		>14
Piperlongumine	1.48	0.95		6 #		>3
IK Erastin	1.49 ***	0.96	0.78	18 $$	554	1
YM155	1.68	0.9	0.5	12 $	36	1
Prima-1	1.83	0.97		6 #		>3
MG-132	1.86	0.91		6 #		>2
Bortezomib	2.07	0.97	0.69	23 ##	5	1

Supplementary Table S3

Confirmation of drug sensitivities predicted by biomarker expression. Cells were treated with a concentration series of drugs for 72 h (exception: DKFZ-682 for 24 h) and the cell viability was quantified by the CellTiter-Blue assay. EC50 values were determined from dose - response curves using GraphPad Prism. The table summarizes the data of at least two independent experiments.

* ratio of EC50 observed in top 3 resistant versus top 3 sensitive cell lines

** H1693 and H661 cells were co-incubated with CM-H2DCFDA fluorescent dye and drug (20 µM DKFZ-682, 100 µM Necrosulfonamide, 10 µM Methylstat, 20 µM 1S3R-RSL3, 20 µM Piperlomgumine, 100 nM IK Erastin, 200 nM YM155, 600 µM Prima-1, 50 µM MG-132, 100 nM and 100 µM Bortezomib) for 30 min and analysed by flow cytometry. Results are shown as fold change compared with CM-H2DCFDA stained but untreated control.

*** distance refers to erastin (CTRP)

\# H23, H661, H1693, H838, H1793, H1944

$ NCIH1299, NCIH522, NCIH2009, HCC827, NCIH1781, NCIH441, NCIH1437, NCIH1573, NCIH2030, NCIH1651, NCIH2228, NCIH2023

§ NCIH2122, NCIH1944, NCIH1793, LXF289, NCIH2023, NCIH2228, NCIH1651, NCIH2030, NCIH1573, NCIH1395, A549, NCIH2126, NCIH1437, NCIH838, NCIH441, HCC827, NCIH661, NCIH2009, NCIH1693, NCIH1568, NCIH2405, NCIH23, NCIH522, NCIH1299

\## NCIH2122, NCIH1944, NCIH1793, LXF289, NCIH2023, NCIH2228, NCIH1651, NCIH2030, NCIH1573, A549, NCIH2126, NCIH1437, NCIH838, NCIH441, HCC827, NCIH661, NCIH2009, NCIH1693, NCIH1568, NCIH2405, NCIH23, NCIH522, NCIH1299

$$ NCIH2122, NCIH1944, NCIH1793, NCIH2126, NCIH1395, LXF289, NCIH1568, NCIH2405, NCIH1651, NCIH1573, A549, NCIH23, NCIH838, NCIH1781, NCIH661, HCC827, NCIH522, NCIH1693

Cell line	IKE EC50 (µM)	DKFZ-682 EC50 (µM)	*AIFM2*	avg ACB	avg ferroptosis defense genes *	*GPX4*
**NCIH2122**	200.00	12.49	5.41	7.41	7.81	5.52
**NCIH1944**	100.00	12.96	5.57	7.22	8.31	6.50
**NCIH1793**	50.00	10.04	4.64	7.24	7.98	7.58
**NCIH2126**	33.84	3.62	3.93	4.52	5.51	5.36
**NCIH1395**	20.35	3.15	3.11	3.90	4.38	4.72
**LXF289**	3.68	7.44	4.98	6.04	7.01	6.08
**NCIH1568**	2.58	1.37	4.46	3.41	4.43	6.04
**NCIH2405**	2.37	1.59	4.19	5.29	5.68	5.76
**NCIH1651**	1.33	2.70	4.25	5.23	6.62	6.77
**NCIH1573**	1.02	7.62	4.90	6.96	6.94	6.80
**A549**	1.01	4.24	4.64	6.61	7.56	5.78
**NCIH23**	0.34	0.94	1.54	2.70	3.70	7.93
**NCIH838**	0.29	2.32	4.46	4.83	6.20	7.46
**NCIH1781**	0.20	1.69	3.52	4.31	5.05	6.86
**NCIH661**	0.19	1.52	2.74	3.22	3.77	6.90
**HCC827**	0.13	1.43	0.99	3.24	4.00	5.91
**NCIH522**	0.07	0.73	2.61	3.10	3.85	6.66
**NCIH1693**	0.03	1.04	3.08	3.73	4.33	7.18
correlation IKE/DKFZ-682		0.78				
correlation EC50 IKE/gene expression			0.47	0.57	0.54	-0.26

Supplementary Table S4

ACB set of biomarkers demonstrates high correlation with sensitivity to ferroptosis-inducing drug IK Erastin (IKE).

***** *GSR, AIFM2, TXN, GCLM, AKR1C3, SLC7A11*

Supplementary Table S5: Primer for qPCR

Target	Primer forward 5´- 3´	Primer reverse 5´- 3´
AKR1C3	CCGAAGCAAGATTGCAGATGGC	GTGAGTTTTCCAAGGCTGGTCG
BLVRB	CTCATGGTGTGGACAAGGTCGT	CATCACAGCCACGTACTTCAGG
CBR1	CTGATCCCACACCCTTTCAT	TTAAGGGCTCTGACGCTAT
GAPDH	TGCGACTTCAACAGCAACTC	CTTGCTCAGTGTCCTTGCTG
GCLM	TCTTGCCTCCTGCTGTGTGATG	TTGGAAACTTGCTTCAGAAAGCAG
GSR	TCACCAAGTCCCATATAGAAATC	TGTGGCGATCAGGATGTG
NRF2 (NFE2L2)	CACATCCAGTCAGAAACCAGTGG	GGAATGTCTGCGCCAAAAGCTG
PGD	GTTCCAAGACACCGATGGCAAAC	CACCGAGCAAAGACAGCTTCTC
PTGR1	GGAAAAGCTGCTGACAGAGTGG	CACTGTTTCTCCACCCTTCACAC
SLC7A11	TCCTGCTTTGGCTCCATGAACG	AGAGGAGTGTGCTTGCGGACAT
STAT1	AAGGGGCCATCACATTCACAT	TCTCAGCAGCCATGACTTTGT
STAT3	GTAGCGCTGCCCCATACC	GGCAGGTCAATGGTATTGCT
STAT5A	TGGAGGACTACAGTGGCCTG	TGTGGTGCTTCTTCAACACCT
STAT5B	TCTACGTGTTTCCTGATCGGC	GCTTGATCTGTGGCTTCACG
TXN (TRX)	GTTGACTTCTCAGCCACGTG	TCACCCACCTTTTGTCCCTT
UGDH	TGTGATGGTGCCCATGCTGTTG	GTCCATCGAAGATAAAGGCTGGC

Supplementary Table S6: Oligos

sgRNA name	sense oligo
CBR1-1_fw	CACCGTCCCCACCGGAACCTCGCCG
CBR1-1_rev	AAACCGGCGAGGTTCCGGTGGGGAC
CBR1-2_fw	CACCGTCGCCGGGGTGCGGAGCAGG
CBR1-2_rev	AAACCCTGCTCCGCACCCCGGCGAC
CBR1-3_fw	CACCGCGGCCGGGCGTGTAACCCA
CBR1-3_rev	AAACTGGGTTACACGCCCGGCCGC
GCLM-1_fw	CACCGCCGTTACTCATCCCGCGGCG
GCLM-1_rev	AAACCGCCGCGGGATGAGTAACGGC
GCLM-2_fw	CACCGAACCGTTACTCATCCCGCGG
GCLM-2_rev	AAACCCGCGGGATGAGTAACGGTTC
GCLM-3_fw	CACCGCCCGCCGCGGGATGAGTAA
GCLM-3_rev	AAACTTACTCATCCCGCGGCGGGC
GSR-1_fw	CACCGCCGCTAGGCAAGACCCAGG
GSR-1_rev	AAACCCTGGGTCTTGCCTAGCGGC
GSR-2_fw	CACCGCGCATGCTTAGTCACCGTG
GSR-2_rev	AAACCACGGTGACTAAGCATGCGC
GSR-3_fw	CACCGCCGCCGCTAGGCAAGACCC
GSR-3_rev	AAACGGGTCTTGCCTAGCGGCGGC
Non-targeting-2_fw	CACCGTTCGTGGTAGGTATAACTAT
Non-targeting-2_rev	AAACATAGTTATACCTACCACGAAC
Non-targeting-3_fw	CACCGTGGTAGTGAGAAGTACTAG
Non-targeting-3_rev	AAACCTAGTACTTCTCACTACCAC
PTGR1-1_fw	CACCGTTCCTCCTGTGACCCTTTCG
PTGR1-1_rev	AAACCGAAAGGGTCACAGGAGGAAC
PTGR1-2_fw	CACCGCCCAGGCAGTGGAACCTTC
PTGR1-2_rev	AAACGAAGGTTCCACTGCCTGGGC
PTGR1-3_fw	CACCGGCACTGCGGGCAAGCGGCA
PTGR1-3_rev	AAACTGCCGCTTGCCCGCAGTGCC
TXN-1_fw	CACCGTCCCGGCTCGCAGGCTCCAG
TXN-1_rev	AAACCTGGAGCCTGCGAGCCGGGAC
TXN-2_fw	CACCGCGGGGCTGGCGCAAGCGTG
TXN-2_rev	AAACCACGCTTGCGCCAGCCCCGC
TXN-3_fw	CACCGAGCCTGCGAGCCGGGATCG
TXN-3_rev	AAACCGATCCCGGCTCGCAGGCTC
UGDH-1_fw	CACCGCTCCCCCTCCCGCTACACA
UGDH-1_rev	AAACTGTGTAGCGGGAGGGGGAGC
UGDH-2_fw	CACCGAGCTGAGGGCAGAATCCAGG
UGDH-2_rev	AAACCCTGGATTCTGCCCTCAGCTC
UGDH-3_fw	CACCGGGAGCTAGGAAGCAGCTGA
UGDH-3_rev	AAACTCAGCTGCTTCCTAGCTCCC
