# Supplementary material for: Low level of antioxidant capacity biomarkers but not target overexpression predicts vulnerability to ROS-inducing drugs
Source: Redox Biol. 2023 Feb 23;62:102639. doi: 10.1016/j.redox.2023.102639 (PMC10053401; doi:10.1016/j.redox.2023.102639)
Supplement: Multimedia component 4 [file mmc4.docx]

**Supplementary Figure S1**

**Inhibitory activities of DKFZ-682 and its analog Auranofin in cell culture assays. (A)** Various concentrations of DKFZ-682 or auranofin were prepared in medium containing 0, 15 or 30 mg/mL additional BSA. After 1 h preparation of solutions, H838 cells were treated for 8 h in 96-well plates with the dilution series of each compound. After washing with fresh medium, inhibitor free medium was added. The numbers of surviving cells were quantified 64 h later using the CellTiter-Blue assay. EC50 values were determined from dose-response curves using GraphPad Prism. **(B)** Three-dimensional (3D) cell spheroids (H1793 and A549 resistant to DKFZ-682 shown in black; H661 and H522 sensitive to DKFZ-682 shown in blue) were treated with a concentration series of DKFZ-682 for 24 h and the cell viability was quantified by the CellTiter-Glow 3D assay. EC50 values were determined from dose-response curves using GraphPad Prism. The graph is representative of two independent experiments each performed in triplicates (error bars indicate SD). For comparison of DKFZ-682 activity in 2D monolayer and 3D spheroid model (right panel), 2D EC50 data of Fig. 1D were used.

**Supplementary Figure S2**

**Redox buffer capacity of ROS drug resistant versus sensitive cells. (A)** Scatter plot of DKFZ-682 EC50 (ln, µM) versus relative levels of basal ROS/RNS. Correlation is assessed by Pearson coefficient on n=31 NSCLC cell lines. NSCLC cell lines were treated with a concentration series of DKFZ-682 for 24 h and the cell viability was quantified by the CellTiter-Blue assay. EC50 values were determined from dose-response curves using GraphPad Prism. For ROS/RNS detection cells were stained with CM-H2DCFDA (ONOO^-^, HO**^.^**), DAF-FM Diacetate (NO), DAX-J2™ PON Green (ONOO^-^), OxiVision^TM^ Green peroxide sensor (H_2_O_2_) or ROS Brite^TM^ 570 (O_2_**^.^**^-^, HO**^.^**) fluorescent dyes and analysed by flow cytometry. **(B)** NSCLC cells were stained with OxiVision^TM^ Green peroxide sensor for 20 min and treated with or without H_2_O_2_ (100 µM) for further 10 min without medium change, and analysed by flow cytometry. The graphs summarize the relative data of independent experiments (n=3-4, error bars indicate SEM). **(C)** Cells were incubated with the indicated concentration of DKFZ-682 for 3 h. Oxidized (ox) and reduced (red) status of PRDX1 and PRDX3 proteins were analysed by immunoblotting. Bar diagrams summarize the quantitative results from independent experiments (n=2-3, error bars indicate SD; **q*<0.05, ***q*<0.01, ****q*<0.001, *****q*<0.0001, two-tailed unpaired *t* test using the original data).

**Supplementary Figure S3**

**Nitric oxide (NO) increase does not reduce DKFZ-682 toxicity.** H23, H661 and HCC827 cells were treated with a concentration series of DKFZ-682 for 24 h in the presence of NO donor NOC-18 (200 µM), and the cell viability was quantified by the CellTiter-Blue assay. Data points represent results from three technical replicates. The results are representative of two independent experiments.

**Supplementary Figure S4**

**ACB protein expression in NSCLC cell lines. (A)** Protein levels of NRF2 and selected ACBs in total cell extracts from 31 NSCLC cell lines were analysed by immunoblotting. Heatmap with the expression values of NRF2 and 8 ACB genes. First protein expression in H1944 was set to 1 for each gene and for each experiment (n=2-6). Then the data were normalized for each gene separately. The smallest value of row was set to 0 %, and the sum of all values in the row was set to 100 %. **(B)** NRF2 is predominantly located in the nucleus, independent of its expression levels. NRF2 protein level in cytoplasmic (C) and nuclear (N) fractions of different NSCLC cell lines were analysed by Western blotting. 60 µg of nuclear and 60 µg of cytoplasmic extracts were used. Lamin A/C and β-tubulin were used as controls for the nuclear and cytoplasmic sub compartments.

**Supplementary Figure S5**

**Overexpression of single ACB genes using CRISPR activation technology. (A)** ACB transcript and protein levels in H23 and HCC827 wild type (WT) or CRISPRa cell lines expressing an enzymatically inactive Cas9 protein (dCas9, only RNA binding activity) with linked transcriptional activators VP64 alone (dCas9/VP64), or with target gene specific (sgRNA) or non-targeting (NT sgRNA) small guide RNAs were analysed by qPCR and immunoblotting respectively. Bars represent mean ± SD of three technical replicates. Transcript level of each gene was normalized to *GAPDH* level. Protein and transcript levels of H1944 were used as control. **(B)** H23 and HCC827 CRISPRa cell lines with non-targeting (NT sgRNA) small guide RNAs or overexpressing *GSR*, *PTGR1*, *CBR1*, *TXN*, *GCLM* or *UGDH* (target gene sgRNA) were treated with a concentration series of DKFZ-682 for 24 h and the cell viability was quantified by the CellTiter-Blue assay. Three technical replicates were performed. EC50 values were determined from dose-response curves using GraphPad Prism. The results of DKFZ-682 EC50 ratio of cells with upregulated gene (target gene sgRNA) and control cells (NT sgRNA) are shown in the table.

**Supplementary Figure S6**

**Knockdown of *NRF2* decreases ROS protection efficiency of DKFZ-682 resistant cells. (A)** H1944 and H1793 cell lines were transfected with nonsense or *NRF2* specific siRNA. The ACB gene expression 48 h after siRNA transfection was quantified using the Affy Clariom S Human array. Expression data derived from *NRF2* knockdown in A549 cells are derived from GSE38332 (GEO accession number). Fold change reduction was calculated as a ratio of three biological replicates each of cells transfected with nonsense or *NRF2* siRNA. The dotted line indicates relative ACB expression in control cells transfected with nonsense siRNA. **(B)** Cell lines were transfected with nonsense (*ctrl*) or *NRF2* siRNA for 48 h and then treated with the indicated concentration of DKFZ-682 for 3 h. Oxidized (ox) and reduced (red) levels of PRDX1 and PRDX3 proteins were analysed by immunoblotting. Representative western blots of at least 2 independent experiments are shown. **(C)** H1944, A549, H1793 and H661 cell lines were transfected with nonsense (ctrl) or *NRF2* specific siRNA. After 48 h cells were treated with a concentration series of DKFZ-682 for 24 h and the cell viability was quantified by the CellTiter-Blue assay. Bar diagrams show the mean of EC50 data from independent experiments (n=4 H1944 and A549, n=2 H1793 and H661) each performed in triplicates (error bars indicate SD, **q*<0.05, ****q*<0.001, *****q*<0.0001, two-tailed unpaired *t* test). NRF2 protein expression was analysed by immunoblotting. Representative western blots are shown.

**Supplementary Figure S7**

**An impact of NRF2 overexpression in DKFZ-682 sensitive cells on redox buffer capacity. (A)** HCC827, H522, H23 and H1693 cell lines were treated with CPUY192018 (30 µM) or DMSO for 6 h**.** *SRXN1* transcript level analysis was performed using qPCR assay. *SRXN1* expression in DMSO treated cells was set to 1. Relative data represent mean of independent experiments each performed in triplicates (n=2, error bars indicate SD). **(B)** HCC827, H23 and H522 cell lines were treated with DMSO or CPUY192018 (10 µM). After 24 the cell medium was changed and cells were treated with DKFZ-682 for 3 h. Oxidized (ox) and reduced (red) levels of PRDX1 protein were analysed by immunoblotting. Bar diagrams show the quantitative results only for prominent DKFZ-682 concentration different for each cell line (n=2, error bars indicate SD). **(C)** Volcano plot (left panel) of genes significantly up- or downregulated in H522 and HCC827 cell lines after 6 h treatment with CPUY192018 (10 µM) or DMSO. Transcript data analysis using RNAseq. An overview of differentially expressed ACB genes under CPUY192018 application in HCC827 and H522 cell lines (right panel). Results are representative of three independent experiments each performed in triplicates (error bars indicate SD, **q*<0.05, unpaired multiple *t* test, comparison DMSO versus drug treatment).

**Supplementary Figure S8**

**ACB expression remains repressed after treatment with DNMT-, HDAC- or HMT inhibitors**. **(A)** Volcano plot shows the results of correlation analysis between methylation values of transcription start sites CpG clusters (reduced-representation bisulfite sequencing data) and EC50. **(B)** H23, H1693 and H522 cells were treated with CPUY192018 (CPUY, 10 µM) after 3 days of culture with or without azacytidine (AZA, 2.5 µM) added on day 1 and 2 after seeding (day 0). After 24 h, cells were treated with a range of concentrations of DKFZ-682 for 24 h. Cell viability was assessed using CellTiter-Blue assay. Bar diagrams represent mean ± SD of independent experiments (n=2-3, ***q*<0.01, ****q*<0.001, *ns*, not significant, paired Student *t* test using the original data). **(C)** HCC827 and H522 cells were treated for 24 h with HDAC inhibitors (5 µM BML-210, 5 µM PAOA or 5 µM BRD-6929) or for seven days with G9a inhibitors (2 µM UNC0642 or 2 µM BIX01294). After this period, CPUY192018 (10 µM) was added for 24 h. Next, cells were treated with series dilutions of DKFZ-682, and after 24 h, cell viability was quantified by the CellTiter-Blue assay. Bars represent results from three technical replicates.

**Supplementary Figure S9**

**NFKB is not required for NRF2-mediated induction of drug resistance. (A)** Gene set enrichment analysis. We performed differential expression analysis between two groups - HCC827, H23 and H522, H1299, H1693, H1781 using limma Bioconductor package. Then, with differential expression results we performed GSEA analysis using as genes sets “transcription factor – genes” association data from Dorothea Bioconductor package. We plotted 10 pathways with highest positive NES and 10 pathways with lowest negative NES from GSEA results. **(B)** Cells were pretreated with DMSO as a control, CPUY192018 (CPUY, 10 µM), SC75741 (1 µM), BAY11-7082 (1 µM), or in combination of CPUY192018 with SC75741 or with BAY11-7082. After 24 h cells were treated with a concentration series of DKFZ-682 for 24 h and the cell viability was quantified by the CellTiter-Blue assay. The results of DKFZ-682 EC50 ratio of DMSO and drug treated cells are shown. Bar diagrams summarize the quantitative results of independent experiments (n=2, error bars indicate SD) each performed in triplicates.

**Supplementary Figure S10**

**Inhibition of STAT activity enhances NRF2 dependent drug resistance. (A)** HCC827 cells were treated with DMSO as a control, TPCA-1 (1 µM), P6 (1 µM) und C188-9 (10 µM) alone or in the presence of CPUY192018 (10 µM) for 6 and 24 h. STAT protein expression was analysed by immunoblotting. Representative western blots of two independent experiments are shown. **(B, C, D, F)** Cells were treated with DMSO as a control, 10 µM C188-9 **(B, D)**, 1 µM P6 **(C),** 1 µM TPCA-1 **(D, F)**, alone or in the presence of CPUY192018 (CPUY, 10 µM) for 24 h. **(B, C)** After pretreatment with indicated compounds cells were treated with series dilutions of DKFZ-682, and after 24 h, cell viability was quantified by the CellTiter-Blue assay. **(D)** Then the cell medium was changed and cells were treated with DKFZ-682 (5 µM or 10 µM) for 3 h. Oxidized (ox) and reduced (red) levels of PRDX1 protein were analysed by immunoblotting. Ratio of oxidized and reduced PRDX1 in control cells was set to 1. Bar diagrams show the mean of EC50 data of independent experiments (n=3 **(B-D)**, n=2-3 **(F)**) each performed in triplicates (error bars indicate SD (**B, C, F**) or SEM (**D**), **q*<0.05, ***q*<0.01, ****q*<0.001, *ns*, not significant, two-tailed unpaired *t* test). **(E)** Protein levels of total and phosphorylated STATs in total cell extracts from NSCLC cell lines were analyzed by immunoblotting. The blots are representatives of two experiments.

**Supplementary Figure S11**

**Lipid composition and steady state iron content does not correlate with sensitivity to DKFZ-682. (A)** Volcano plot visualizing correlations between lipid levels and EC50 values. Liquid chromatography–mass spectrometry metabolite data. **(B)** DKFZ-682 does not induce ferroptosis, as shown by the lipid peroxidation sensor Bodipy 581/591 C11. After a pre-treatment with ferroptosis inhibitor ferrostatin-1 (Fer-1, 10 µM), cells were stained with Bodipy (3 µM, 3 h) in the presence of either DKFZ-682 (5 µM for H661 and H838, 20 µM for A549 and H1793) or a ferroptosis inducer ML210 (10 µM). After washing, cells were analyzed using a flow cytometer. The ratio of the oxidized (excitation 488 nm, emission 530/30) and reduced (excitation 561 nm, emission 610/20) dye is shown for each condition. **(C)** Flow cytometry labile iron pool assay in the NSCLC panel with FRET Iron Probe 1 (FIP-1) enables ratiometric fluorescence imaging of labile iron pools in living cells. Mean Green/FRET ratio was obtained for each cell line, and the signal was normalized to the cells treated with an iron chelator - deferoxamine (DFO). No significant difference was observed between high and low ACB expressing cell lines. Error bars denote SD, n=3.

**Supplementary Figure S12**

**Coordination expression of ACB proteins in NSCLC tumors.** Correlation plots show protein to protein correlations for ACB genes in 141 NSCLC tumors, derived from proteomics data Lehtio et al. [35].

**Supplementary Figure S13**

**Expression space of ACBs in cell lines, PDX models and LUAD patients. (A)** Principal component analysis plot (1^st^ and 2^nd^ PCA components) on ACB genes. Samples include NSCLC cell lines (blue) and PDX (black) models. Sensitivity of cell lines is indicated by the color code (blue – sensitive, orange – resistant). **(B)** Cell killing effect after 5 days incubation of tumor fragments (black – low ACBs, red – high ACBs) with DKFZ-682. Cell viability was quantified with CellTiter-Glo. Data are presented as the mean ± SD of six technical replicates. **(C)** Heatmap with ACB genes expression in cell lines TCGA-LUAD tumor and control samples, and PDX models. Rows (genes) are clustered using “complete linkage” method and “Euclidean” distance. Top annotation includes EC50 values (for cell lines) and sample type.

**Supplementary Figure S14**

**(A) Comparison of average ACB expression in AML samples from the TCGA dataset and AML cell lines from CCLE.** The red dashed line indicates ACB expression in the NSCLC line H23. Blue symbols show cell lines included in the AML cell panel used to determine the activity profile of DKFZ-682. Green lines represent the mean ACB value for each dataset. Cell line data are in log_2_(TPM+1) units. TCGA-LUAD expression data were converted from RPKM to log_2_(TPM+1) units. **(B)** Scatter plot of DKFZ-682 EC50 (ln, µM) versus ACB_(11)_ (11 of 15 selected ACB genes) expression. The Pearson correlation of 11 ACB expression and drug sensitivity within the AML cell lines panel (n=17) is r=0.69, p=0.002. AML cell lines were treated with a concentration series of DKFZ-682 for 24 h and the cell viability was quantified by the CellTiter-Blue assay. EC50 values were determined from dose-response curves using GraphPad Prism. Average expression of 11 selected ACBs (see supplementary information (source data) for figure 8B) is based on CCLE data.
