# Supplementary material for: Low level of antioxidant capacity biomarkers but not target overexpression predicts vulnerability to ROS-inducing drugs
Source: Redox Biol. 2023 Feb 23;62:102639. doi: 10.1016/j.redox.2023.102639 (PMC10053401; doi:10.1016/j.redox.2023.102639)
Supplement: Multimedia component 5 [file mmc5.pdf]

Materials and Methods (supplementary)

CellTiter-Glo 3D cell viability assay and determination of EC50

Cells (H1793, A549, H661, H522) were seeded in a Nunclon™ Sphera™ 96-well u-shaped-bottom microplate (Thermo Scientific™, Cat No. 174925) at a density of 2,500 cells/100 µL/well in media containing 10 % FBS (Capricorn scientific), 100 U/mL penicillin, 100 µg/mL streptomycin (Sigma-Aldrich) and centrifuged for 10 min at 400 g. After 72 h, compact spheroids were treated with test compound (all concentrations in triplicate) for 24 h and viable cells were quantified in a FLUOstar OPTIMA ELISA reader 30 min after CellTiter-Glow 3D staining (Promega, Cat No. G9681). Mean values +/- SD were calculated. EC50 were calculated from dose response curves by GraphPad Prism.

RNA isolation and RT-qPCR

Total RNA was isolated using NucleoSpin® RNA Kit according to the protocol (Macherey-Nagel, Cat No. 740955.250). Purified RNA was measured by Nanodrop (Thermo Fisher) and reverse transcription was performed using 1000 ng total RNA using Revert Aid First Strand cDNA Synthesis Kit (Thermo Scientific, Cat No. K1622) and PCR reactions with respective primer pairs were analysed in technical triplicates. Primer sequenes used for quantitative real-time PCR analyses are listed in Supplementary Table S5. *GAPDH* was used for normalization of gene expression.

Cloning of sgRNA vectors for lentiviral particle production

Vectors were cloned, containing three individual small guide RNAs (sgRNA) for the genes *GSR*, *TXN*, *UGDH*, *GCLM*, *CBR1*, *PTRG1*, as well as two non-targeting sgRNAs. Sense and antisense oligo pairs of the sgRNAs (Supplementary Table S6) were phosphorylated and hybridized using a thermocycler (45 min 37 °C, 95 °C 4 min, cool down to 25 °C with a ramp rate of - 0.1 °C / sec): 1 µL sense oligo (100 µM, TE), 1 µL antisense oligo (100 µM, TE), 1 µL T4 DNA-ligase buffer (10 x), 0.5 µL T4 polynucleotide kinase, 6.5 µL H_2O.

To obtain a vector backbone for sgRNA ligation, pXPR_502 (Addgene, Cat No. 96923) was digested using BsmBI (2 µg vector, 2 µL BsmBI-v2 (NEB, Cat No. R0739S), 2.5 µL 10x NEB3.1, up to 25 µL H_2O). Mixture was incubated at 55 °C for 2 h 45 min and was then separated in a 1 % agarose gel (100 V, 45 min). Digestion results in a dropout of 30 bp, the remaining backbone (8664 bp) was extracted from the gel (Zymoclean gel DNA recovery kit, ZYMO RESEARCH, Cat No. D4007). A 1:200 dilution of the hybridized sgRNA oligos and the digested backbone were ligated at RT for 1 h: 50 ng digested and purified pXPR_502 plasmid, 1 µL phosphorylated and annealed oligo pair (1:200 dilution in H_2O), 2 µL T4 DNA-Ligase Buffer (10 x), 0.25 µL T4 DNA-Ligase, up to 20 µL H_2O.

The ligation mix was then used to transform NEB® Stable Competent E. coli (NEB, Cat No. C3040H). Therefore, bacteria were thawed on ice for 10 min, the ligation mix was added, and the cells were incubated for 30-60 min on ice. Next, a heat shock at 42 °C for 45 sec was performed, followed by incubation on ice for 2 min. Then, 250 µL SOC medium was added and the cells were incubated at 37 °C for 1 h to recover. The entire vial of bacteria was plated in ampicillin agar plates (100 µg/mL) and incubated for 12-16 h at 37 °C. The next day, single colonies were picked and used to inoculate small liquid cultures (3 mL LB-Medium, 100 µg/mL ampicillin), incubation at 37 °C for 12-16 h, 220 rpm. Using a miniprep kit (NucleoSpin Plasmid, Macherey-Nagel, Cat No. 740588.250), the vectors were isolated.

To validate the correct insertion, plasmids were sequenced (LightRun Tubes, eurofins) using a U6 sequencing primer (5'-GAGGGCCTATTTCCCATGATTCC-3'). Correct clones were then used for bigger liquid cultures (150 mL LB-Medium, 100 µg/mL ampicillin) and vectors were isolated using a HiSpeed Plasmid Midi Kit (Qiagen, Cat No. 12643).

Lentivirus particle production and transduction of target cells

Polyclonal HCC827and H23 cells constitutively expressing the CRISPR activation machinery were engineered by transducing wild-type cells with lentiviral particles carrying a dCas9-VP64 (lenti dCas9VP64_Blast, Addgene plasmid #61425) at a multiplicity of infection (MOI) of ~0.5. dCas9VP64 expressing cells were then transduce with pXPR_502 carry the sgRNA and transcriptional activation domains p65-HSF1. CRISPRa cell lines were selected for puromycin (4 µg/µL for sgRNAs) and blasticidin (25 µg/µL for dCas9-VP64 construct) every 2-3 weeks for

72 h. Large-scale lentivirus production was performed using a second-generation lentivirus system and a calcium phosphate transfection kit (Invitrogen, Cat No. K278001) in HEK293T cells. Briefly, early passaged HEK293T cells were co-transfected with the lentiviral transfer plasmid, a packaging plasmid (psPAX2, Addgene plasmid #12260), as well as with a plasmid encoding the VSV-G envelope (pMD2.G, Addgene plasmid #12259). Viral supernatant was collected 26-30 h post transfection and stored at -80 °C until use. All experimental procedures for lentivirus production and transduction were performed in a biosafety level 2 laboratory.

Lipid peroxidation

As an indicator of ferroptosis, lipid peroxidation was analyzed in cells stained with Bodipy 581/591 C11 (Invitrogen). Cells were stained with 1.5 µM Bodipy 581/591 C11 diluted in the culture medium for 2 h at 37 °C. Afterwards, cells were detached with TrypLE (Gibco), washed with PBS, and analyzed using the flow cytometer BD LSR Fortessa (BD Biosciences). The ratio of the oxidized (excitation 488 nm, emission 530/30) and reduced (excitation 561 nm, emission 610/20) dye was calculated for each cell in the FlowJo software.

Iron pool assay

FIP-1 probe (FRET Iron Probe) was a gift from Christopher Chang. It enables ratiometric fluorescence imaging of labile iron pools in living cells [73]. Cells were seeded on a 12-well plate to achieve 60-70 % confluence on the day of the assay. 250 µM deferoxamine (DFO) (Sigma) or PBS was added to Fluorobrite media containing 10 % FBS in wells containing cells and incubated at 37 °C for 6 h. After the incubation, media was aspirated and cells were washed with 500 µL HBSS. Then 500 µL HBSS containing 10 µM FIP-1 (diluted from 5 mM stock) was added to each well and this was incubated at 37 °C for 90 min. Cells were harvested by trypsinization, washed once with PBS and FIP-1 fluorescence was analyzed with a flow cytometer (Guava easyCyte 14HT, Luminex) in two channels: Green-B (“Green”, excitation 488, emission 512/18), which is high in the presence of iron, and Yellow-B (“FRET”, excitation 488, emission 575/25), which is low in the presence of iron. Mean Green/FRET ratio was obtained for each cell line, and the signal was normalized to the cells treated with

an iron chelator - deferoxamine (DFO, final concentration 300 μM, 6 h treatment prior to the staining). Unstained cells were used as a control for flow cytometry.
